# Using brefeldin A to disrupt cell wall polysaccharide components in rice and nitric oxide to modify cell wall structure to change aluminum tolerance

**DOI:** 10.3389/fpls.2022.948212

**Published:** 2022-08-05

**Authors:** Jianchao Yan, Jiandong Zhu, Jun Zhou, Chenghua Xing, Hongming Song, Kun Wu, Miaozhen Cai

**Affiliations:** ^1^College of Geography and Environmental Sciences, Zhejiang Normal University, Jinhua, China; ^2^College of Agriculture, Jinhua Polytechnic, Jinhua, China

**Keywords:** aluminum toxicity, nitric oxide, brefeldin A, cell wall polysaccharide, FTIR, functional group

## Abstract

The components and structure of cell wall are closely correlated with aluminum (Al) toxicity and tolerance for plants. However, the cell wall assembly and function construction in response to Al is not known. Brefeldin A (BFA), a macrolide, is used to disrupt cell wall polysaccharide components, and nitric oxide (NO), a signal molecule, is used to modify the cell wall structure. Pretreatment with BFA accelerated Al accumulation in root tips and Al-induced inhibition of root growth of two rice genotypes of Nipponbare and Zhefu 802, and significantly decreased the cell wall polysaccharide content including pectin, hemicellulose 1, and hemicellulose 2, indicating that BFA inhibits the biosynthesis of components in the cell wall and makes the root cell wall lose the ability to resist Al. The addition of NO donor (SNP) significantly alleviated the toxic effects of Al on root growth, Al accumulation, and oxidative damage, and decreased the content of pectin polysaccharide and functional groups of hydroxyl, carboxyl, and amino in the cell wall *via* FTIR analysis, while had no significant effect on hemicellulose 1 and hemicellulose 2 content compared with Al treatment. Furthermore, NO didn't change the inhibition effect of BFA-induced cell wall polysaccharide biosynthesis and root growth. Taken together, BFA disrupts the integrity of cell wall and NO modifies partial cell wall composition and their functional groups, which change the Al tolerance in rice.

## Introduction

Aluminum (Al) is by far the most widespread metallic element within the earth's crust, with only oxygen and silicon in mass (Zhao et al., 2020). When the pH of the soil is lower than 5.0, the insoluble aluminum in the soil is dissolved and released in the form of a highly active aluminum ion such as Al^3+^, various hydroxyl aluminum such as AlOH^2+^, Al(OH)${}_{2}^{+}$, and so on (Singh et al., 2017; Zhao et al., 2020). And only a micromolar concentration of highly active aluminum in acidic soil inhibits plant growth, causes the imbalance of the reactive oxygen species (ROS) system and changes the cell wall structure and function, and eventually leads to the reduction of crop yield. Studies had shown that Al rapidly crosses the plasma membrane and activates the Fenton reaction in the cytoplasm, thereby increasing the content of ROS in cells (Taylor et al., 2000). For example, under Al toxicity, maize (Du et al., 2020), wheat (Yu et al., 2018), black gram (Chowra et al., 2017), rice (Lan et al., 2021), and other plants are induced to produce excessive ROS, which breaks ROS homeostasis and induces oxidative damage in plants (Ranjan et al., 2021b). In addition, Al also increases the contents of pectin and hemicellulose within the root cell wall, and combines with the pectin matrix, further making the cell wall thick and rigid (Nagayama et al., 2019; Zhu et al., 2019), which can also be found in barley (Jaskowiak et al., 2019) and trifoliate orange (Yan et al., 2018). Thus, maintaining the system balance of Al-induced ROS and the stability of cell wall components and structure contribute to the improvement of Al tolerance, and the regulation of signal molecules plays a key role in this process.

Plant root cell wall is a solid matrix wall structure that is made up of protein and polysaccharide biopolymers, which is the first barrier to resist biotic and abiotic stress (Zhang et al., 2020), such as heavy metal toxicity, drought (Zhang et al., 2021b), salinity (Liu et al., 2021), and fungal infection (Cao et al., 2021). It is essential to maintain the integrity and proper structure of cell walls when plant cells, tissues, or whole organs are facing different stresses (Wilmowicz et al., 2022). For Al toxicity, the cell wall of Al-sensitive wheat (Ma et al., 2004), rice (Yang et al., 2008), and citrus (Zhang et al., 2022) species had higher Al retention capacity than Al tolerant species, which is correlated with the modification and composition of cell wall polysaccharides. The modification and composition of cell wall polysaccharides are correlated with Al sequestration in the cell wall. On the contrary, the disruption of polysaccharide compositions in the cell wall is related to Al sensitivity or tolerance in plants. The overexpression of UDP glycosyltransferase genes reduced polysaccharide components in soybean hairy root's cell wall, such as callose and/or hemicellulose and mono-saccharide, then decreased less Al binding in the cell wall (Wang et al., 2021). The increasing accumulation of xyloglucan (XyG), the main component of hemicellulose, or decreasing O-acetylation level of XyG ultimately intensified Al toxicity by affecting the Al-binding capability in *Arabidopsis* (Zhu et al., 2012, 2014). Short-chain aldehydes enhanced the polysaccharide contents and demethylated pectin content in the cell wall of wheat seedlings, increasing Al retention and sensitivity (Liang et al., 2022). The hydrolysis of pectins mediated by MsPG4 enhanced the extensibility of the cell wall and Al tolerance in alfalfa seedlings (Fan et al., 2022). It follows that once the biosynthesis, assembly, and modification of the cell wall are suppressed or disrupted, the power of the plant to resist external stresses is severely weakened.

Brefeldin A (BFA), a macrolide, is usually used as a common tool in the study of eukaryotic membrane vesicle transport (Foissner et al., 2020), i.e., preventing the vesicle trafficking of Golgi which is closely connected with the biosynthesis of cell wall (Zhu et al., 2017). Furthermore, BFA destroys the structure of the Golgi compartments in many types of cells (Li et al., 2019). In recent years, a mass of studies had used BFA to illustrate the cell wall function. For example, after exposure to BFA for 24 h, pectinesterase (PE1), the main enzyme that promotes plant cell wall modification and decomposition, aggregates in vesicle-like structures, suggesting that secretion of vesicles was attenuated by BFA treatment (Wang et al., 2018). A similar phenomenon could be observed in the study of pollen tube (PT) growth. BFA reduced rice PT growth in a concentration-dependent manner (Kim et al., 2021) by inhibiting the secretion of Golgi, and deriving cell wall substances (Grebnev et al., 2017). This is because the cell wall in the top of PT mainly consists of methylated pectin, this pectin is synthesized in the Golgi apparatus and is secreted to the cell surface (Grebnev et al., 2020). These results showed that BFA plays a crucial role in regulating the secretion of cell wall materials. In this study, exogenous addition of BFA was used to produce the defective cell wall suffering from Al toxicity.

Nitric oxide (NO), the multifunctional gaseous signaling molecule, is unique and modulates specific biological functions (Gupta et al., 2022). Previous studies showed that NO is an important signal-regulating molecule in plants, which is not only involved in regulating miscellaneous physiological processes of plants, such as regulating the senescence in plants (Hussain et al., 2022) and the level of cell wall polysaccharide (Sun et al., 2016), but also enhances the plant defense response to various abiotic stresses, heavy metal or such like (Yao et al., 2019). The first proposed role of NO is the regulation of cell wall composition and structure to resist toxic ions such as Al. As early as 2011, it was reported that NO improves the tolerance to Al by affecting the polysaccharides components in the cell wall of rice roots (Zhang et al., 2011). Post-translational modification of proteins, involved in cell signal transduction under stresses (Pande et al., 2022), is another important role of NO in the construction of the functional wall structure. NO regulated the peroxidase (POX), pectin methylesterase (PME), xyloglucan endo-transglycosylases (XET), and other proteins related to cell wall biosynthesis and decomposition (Ruiz-May et al., 2019). Pectic and hemicellulosic compounds usually possess specific acetylation and ethyl esterification profiles *via* the chemical groups, such as methyl, acetyl, and phenyl epitopes, with the changing of physicochemical characteristics. For example, Ca and Si detoxified Al toxicity by increasing the content of cell wall fractions and modifying the carboxyl, cellulose, and esterified pectin with FTIR analysis (Li et al., 2022). Our previous studies also indicated that NO reduced the activity of PME and content of pectic and hemicellulose, then decreased the Al-binding capacity of rice roots (Lan et al., 2021). Although the modification of cell wall polysaccharides to Al accumulation had been explored in previous studies, the central factors involved in the regulatory role of NO leading to cell wall assembly and function construction, and a subsequently decreased Al-binding capacity, remained a mystery under Al toxicity.

In this study, BFA, an inhibitor of Golgi secretion, was used to disrupt the biosynthesis of the cell wall, and the exogenous NO donor or scavenger was used to regulate the modification of cell wall components and functional groups. The present study aimed to investigate (1) the contribution of cell wall integrity on rice Al tolerance, and (2) the role of NO in modifying cell wall structure. These achievements will provide new insight into how NO works in the cell wall assembly and structure conduction in Al tolerance.

## Materials and methods

### Plant materials and treatments

Two rice (*Oryza sativa* L.) genotypes were used for investigation, Nipponbare is an Al-resistant genotype and Zhefu 802 is an Al-sensitive genotype (Yang et al., 2008). Seeds were sterilized with 1% NaClO solution for 20 min and washed with distilled water, then evenly spread in a plastic box which is covered with moistened gauze with sterile water, then placed in an incubator with 80% relative humidity for germination (25°C, dark). After root length reached about 1 cm, seedlings were transferred to the total nutrition solution and cultured to a single leaf. The nutrient solution consists of 1.51 × 10^5^ mg·L^−1^ (NH_4_)_2_SO_4_, 4.03 × 10^4^ mg·L^−1^ NaH_2_PO_4_·2H_2_O, 7.14 × 10^4^ mg·L^−1^ K_2_SO_4_,8.86 × 10^4^ mg·L^−1^ CaCl_2_, 3.24 × 10^5^mg·L^−1^ MgSO_4_·7H_2_O, 1.5 × 10^3^ mg·L^−1^ MnCl_2_·4H_2_O, 74 mg·L^−1^ (NH_4_)_6_·MO_7_O_24_·4H_2_O, 9.34 × 10^2^ mg·L^−1^ H_3_BO_3_, 35 mg·L^−1^ ZnSO_4_·7H_2_O, 31 mg·L^−1^ CuSO_4_·5H_2_O, 7.7 × 10^3^ mg·L^−1^ FeCl_3_·6H_2_O and 1.19 × 10^4^ mg·L^−1^ C_6_H_10_O_8_, (pH 5.0–5.5) (Yoshida et al., 1971). The solutions were changed every 3 days. When the plant height reached about 15 cm, seedlings were transplanted to a 10-L plastic bucket (20 cm × 24 cm × 26 cm). Uniform seedlings were planted in a light chamber with a 14/10 h and 26/23°C day/night light and temperature cycle, the light intensity of 400 μmol photons/m^2^/s, and the relative humidity of 80%. After 7 days of growth, rice seedlings were treated with Al, NO donor, and NO scavenger.

To clarify the role of NO in modifying cell wall structure, we set up five treatments containing 500 μM CaCl_2_ with pH 5.5: (1) Control, (2) Al, (3) Al + SNP, (4) Al + cPTIO, (5) Al + NaN_3_. Sodium nitroprusside (SNP) was used as NO donor, 2-(4-carboxyphenyl)-4,4,5,5-tetramethylimidazoline-1-oxyl-3-oxide (cPTIO) was applied as NO scavenger and NaN_3_ was applied as endogenous nitrate reductase (NR) inhibitor. Rice seedlings were, respectively, pretreated with 100 μM SNP, 75 μM cPTIO, and 5 μM NaN_3_ for 12 h with 500 μM CaCl_2_ solution, then transfer to 50 μM AlCl_3_ for 24 h. The control and Al groups were pretreated with 500 μM CaCl_2_ for 24 h, and the seedlings of the Al group were treated with 50 μM AlCl_3_ for 24 h. After that, rice was collected and cleaned with 500 μM CaCl_2_ solution for 30 min to elute the Al^3+^ on the root surface, then rinsed with distilled water. The oxidative damage and functional groups of the cell wall were measured.

To verify the importance of cell wall integrity for aluminum tolerance and the regulator for the biosynthesis of the cell wall in rice, BFA was used to disrupt cell wall polysaccharide components and SNP was applied as the regulator for the polysaccharide structure of cell wall. We set up seven treatments: (1) Control, (2) Al, (3) SNP, (4) BFA, (5) SNP + Al, (6) BFA + Al, (7) SNP + BFA + Al. Rice seedlings were pretreated with 250 μM SNP or/and 0.5 μM BFA (in 500 μM CaCl_2_ solution, pH 5.5) for 24 h before transferred to 50 μM AlCl_3_ (in 500 μM CaCl_2_ solution, pH 5.5) for 24 h. The control and Al groups were pretreated with 500 μM CaCl_2_ for 24 h, after that the seedlings of the Al group were treated with 50 μM AlCl_3_ (in 500 μM CaCl_2_ solution, pH 5.5) for 24 h. The plant growth and cell wall composition were measured.

### Determination of root elongation

Rice root elongation was measured with a ruler before and after SNP, cPTIO, NaN_3_, BFA, and Al treatment. Relative root elongation is determined by the ratio of root elongation in other treatment groups to that in the control group.

### Hydrogen peroxide (H_2_O_2_), superoxide free radical (O^2–^), and lipid peroxidation determination in root tips and *in situ* observation

The content of H_2_O_2_ in root tips was determined by the xylenol orange method (Gay et al., 1999). Root tips (30 apexes per sample) were ground in pre-cooled acetone, then the solution was centrifuged at 10,000 × *g* for 20 min. The H_2_O_2_ in the supernatant was analyzed and calculated by the H_2_O_2_ standard curve.

The production rate of O^2−^in root tips was determined by the hydroxylamine method (Eticha et al., 2005). The root tips (30 apexes per sample) were ground in pre-cooled 2 mL of 65 mM PBS (pH 7.8). The solution was centrifuged at 6,000 × *g* for 15 min at 4°C. Finally, the O^2−^in the supernatant was measured at 530 nm using a UV spectrophotometer.

For the determination of MDA, the method of thiobarbituric acid colorimetry was used (Su et al., 2019).

Hydrogen peroxide (H_2_O_2_), O^2−^, and lipid peroxidation *in situ* observation. For H_2_O_2_ detection, root tips with 1-cm long were incubated for 20 min at 37°C, with 10 μM 2',7'-dichlorofluorescein diacetate (DCFH-DA) prepared in 50 mM PBS (pH 7.4). O^2−^was detected by staining with a 10 μM superoxide anion fluorescent probe (dihydroethidium, DHE) for 30 min at 37°C. Then, the root tips were washed three times in the buffer of 50 mM PBS (pH 7.4). For lipid peroxidation detection, root tips were incubated in 0.025% Evans blue solution (with 100 mM CaCl_2_, pH 5.6) for 10 min, then root tips were washed with 100 mM CaCl_2_ (pH 5.6) three times to protect the dye from being eluted. Root tips were observed in the fluorescence microscope (Yamamoto et al., 2001; Lv et al., 2017).

### Determination of mononuclear Al, Al content in root tips and cell wall

For mononuclear Al analysis, roots were cleaned with 0.5 mM CaCl_2_, and quickly put into a syringe and pressed to obtain root cell sap, then added to 400 μl of 1 M NH_4_OAc-HOAc buffer (pH 4.0), further added 800 μl of Morin (1.0 × 10^−4^ M) dissolving in ethanol, and finally add ethanol to make the total volume reach 4 ml. The mononuclear Al was determined by the fluorescence spectrophotometer (403 nm excitation, 490 nm emission) (Stass et al., 2006).

For determination of Al content in the cell wall, the root tips (30 apexes per sample) were extracted in 1 ml of 1 M HCl for 24 h, and the Al content was determined by the inductively coupled plasma spectrometer (ICP) method (Liao et al., 2006).

For determination of Al content in the cell wall, the pre-extracted cell wall was added to 1 ml 2 M HCl and suspended at room temperature for 24 h (occasional shaking) according to the study by Yang et al. (2008) and the Al content was determined by ICP method.

After 24 h Al treatment, fresh roots were manually cut for *in situ* observation. The fluorescence detection methods refer to Gunsé et al. (2000). The 1-cm-long root tip was immersed in 5 mM NH_4_OAC buffer (pH 5.0 with acetic acid) for 5 min, transferred to 100 μM Morin solution (containing 5 mM NH_4_OAC, pH 5.0) for 1 h, and then immersed in 5 mM NH_4_OAC buffer for 10 min. The fluoresce green was observed in the fluorescence microscope.

### NO determination in root tips

The production of NO was determined as described by Murphy and Noack (1994). Rice root tips (0.2 g) were added to 100 units of catalase (CAT) and 100 units of superoxide dismutase (SOD) for 5 min to eliminate endogenous ROS. Then, add 10 ml of 5 mM oxyhemoglobin to root tips at room temperature, and incubate the mixture for 5 min. Finally, measuring the absorbance at 401 and 421 nm spectrophotometrically.

### Extraction of root cell wall

We extract cell walls by employing 30 fresh rice root tips with the method reported by Zhong and Lauchli (1993). After grinding root tips with 0.5 ml of cold deionized water until homogenous, then centrifuged (4°C, 10,000 rpm) for 10 min. The resulting precipitate was washed twice with 1 ml 80% ethanol, once with 1 ml methanol: chloroform (1:1 [V/V]) solution and 1 ml acetone, respectively. After freeze-drying, the required cell wall is formed.

### Determination of cell wall components

The cell walls obtained by extraction were added to 0.75 ml of 0.5% ammonium oxalate buffer solution, then placed in boiling water (lasted 1 h, repeated twice). Centrifuge the mixed solution (12,000 rpm) for 10 min, and the supernatant is the pectin solution. Then, 0.75 ml 4% KOH was added to the precipitate (lasted 12 h), centrifuged (12,000 rpm) for 10 min (repeated two times), and the supernatant obtained was hemicellulose 1. The extraction process of hemicellulose 2 was analogous, but the 24% KOH solution was used.

The content of uronic acid was determined by the method reported by Blumenkrantz and Asboe-Hansen (Blumenkrantz and Asboe-Hansen, 1973), and used this to express the content of pectin. As previously reported, hemicellulose content was determined based on total sugar content (Dubois et al., 1956).

### Fourier-transform infrared spectroscopy (FTIR) of root cell wall

The functional groups on the cell wall were determined by FTIR (Nexus 670 of Thermo Nicolet from the United States). The freeze-dried root cell wall (1 μg) was crushed with 150 mg of potassium bromide (KBr), then pressed into thin slices. Functional groups were identified using FTIR in the wavelength range of 400–4,000 cm^−1^ with a resolution of 4 cm^−1^ and performed 64 scans.

### Statistical analysis

SPSS (version 21.0.0) software was used for statistical analysis of experimental data, and ANOVA (analysis of variance, Tukey's posttest) was used for the significant difference test (*P* < 0.05).

## Results

### SNP donor increased NO production and excluded mononuclear Al in root tips

**Figure 1:**
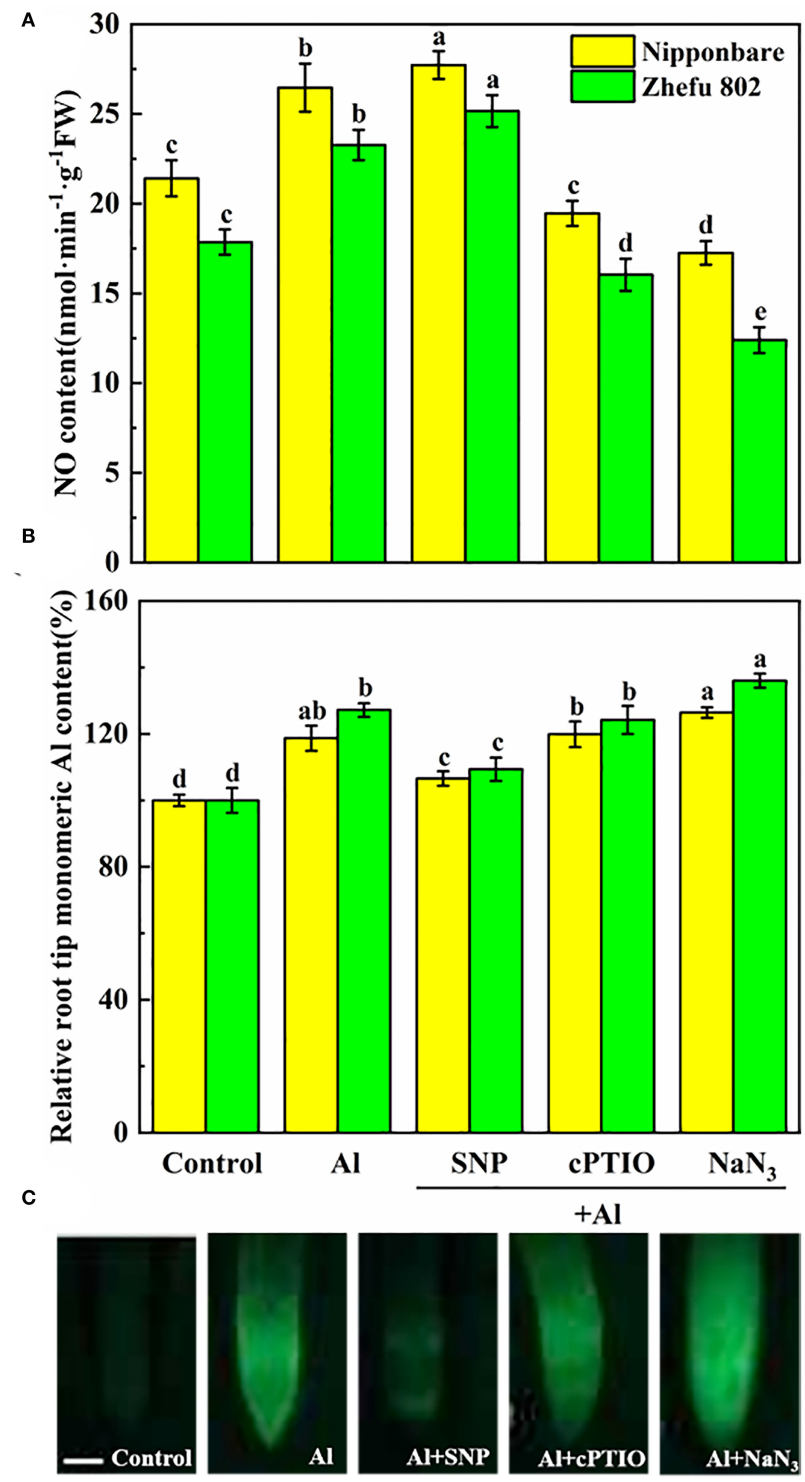
Effect of Al toxicity and SNP donor on **(A)** endogenous NO content, **(B)** mononuclear Al content, and **(C)** Al accumulation by Morin fluorescence staining in rice root tips. Columns with different letters are significantly different at *P* < 0.05. Bar in graph C indicated 200 μm.

To investigate the response of endogenous NO biosynthesis to Al toxicity in rice root tips, the SNP (NO donor), cPTIO (NO scavenger), and NaN_3_ (NR inhibitor) were applied to measure the NO production. To affirm that the induction effect of SNP supplementation caused the NO production, 75 μM cPTIO and 5 μM NaN_3_ were treated with Al. Compared to Al-treated rice plants, the endogenous NO level was decreased by 25.3 and 31.1% treated with cPTIO in Nipponbare and Zhefu 802 (Figure 1A). The NR inhibitor NaN_3_ also significantly inhibited the production of NO in the roots of two rice genotypes. Furthermore, the inhibitory effects of cPTIO and NaN_3_ were reversed by SNP addition, suggesting that the NR-dependent NO production involved Al toxicity.

As shown in Figure 1B, mononuclear Al content increased by 18.7 and 27.2% in root tips of Nipponbare and Zhefu 802 after 24 h of treatment. After SNP treatment, mononuclear Al in Nipponbare and Zhefu 802 decreased by 10.2 and 14.0%, respectively, compared to Al treatment. Inhibition of NO production by cPTIO and NaN_3_ reversed the role of NO on Al accumulation. We also used Morin staining as an indicator for measuring Al accumulation (Figure 1C). The result of Morin staining indicated SNP strongly reduced Al accumulation, and the green fluorescence is very weak.

### SNP donor protected oxidative damage in root tips of rice

**Figure 2:**
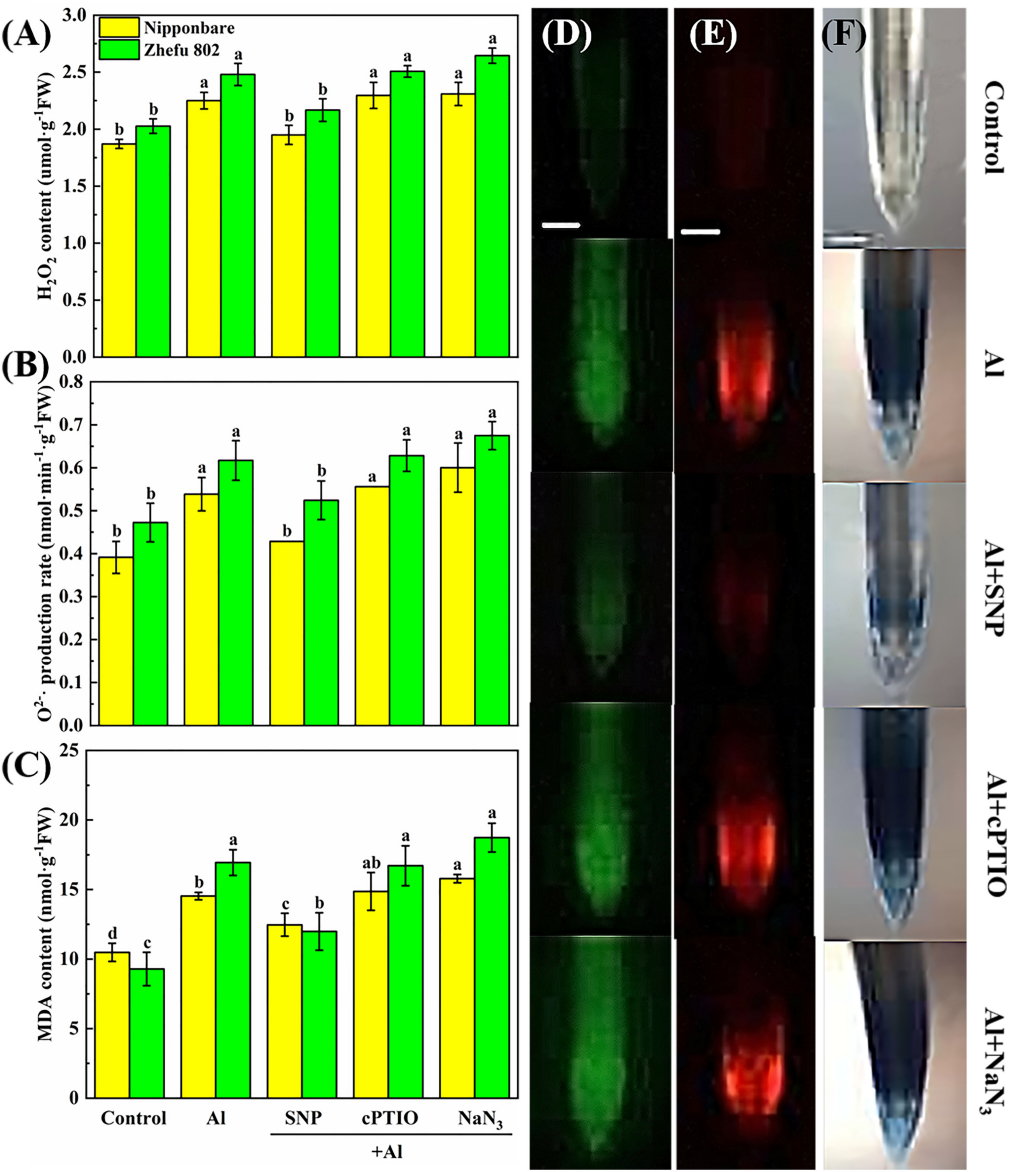
The effect of SNP and NO scavengers on **(A)** H_2_O_2_, **(B)** O^2−^·production rate, and **(C)** MDA in rice root tips under Al toxicity and **(D)** H_2_O_2_, **(E)** O^2−^, and **(F)** oxidative damage *in situ* observation in root tips. Columns with different letters are significantly different at *P* < 0.05. Bar in graph **(D–F)** indicated 200 μm.

To clarify the protective effect of NO on Al toxicity, the accumulation of H_2_O_2_, O^2−^, and MDA was analyzed, and DCFH-DA, DHE, and Evans blue dye solution were used to detect H_2_O_2_, O^2−^, and oxidative damage *in vivo*, respectively. Al treatment significantly enhanced the production of H_2_O_2_, O^2−^, and MDA. Upon supplementation of SNP, the accumulation of H_2_O_2_, O^2−^, and MDA reduced markedly (Figures 2A–C). Whereas, the application of the cPTIO and the NaN_3_ increased the H_2_O_2_, O^2−^, and MDA levels. Homoplastically, the results of the fluorescence probe of DCFH-DA and DHE indicated that the rice roots showed strong fluorescence brightness in the roots treated with Al, cPTIO, and NaN_3_, and showed weak fluorescence brightness in the roots treated with SNP (Figures 2D,E). Similar results were obtained by Evans blue staining (Figure 2F). The results indicated that NO has a protective effect on oxidative damage caused by Al.

### SNP regulated the functional groups of the cell wall in rice root tips with FTIR analysis

**Figure 3:**
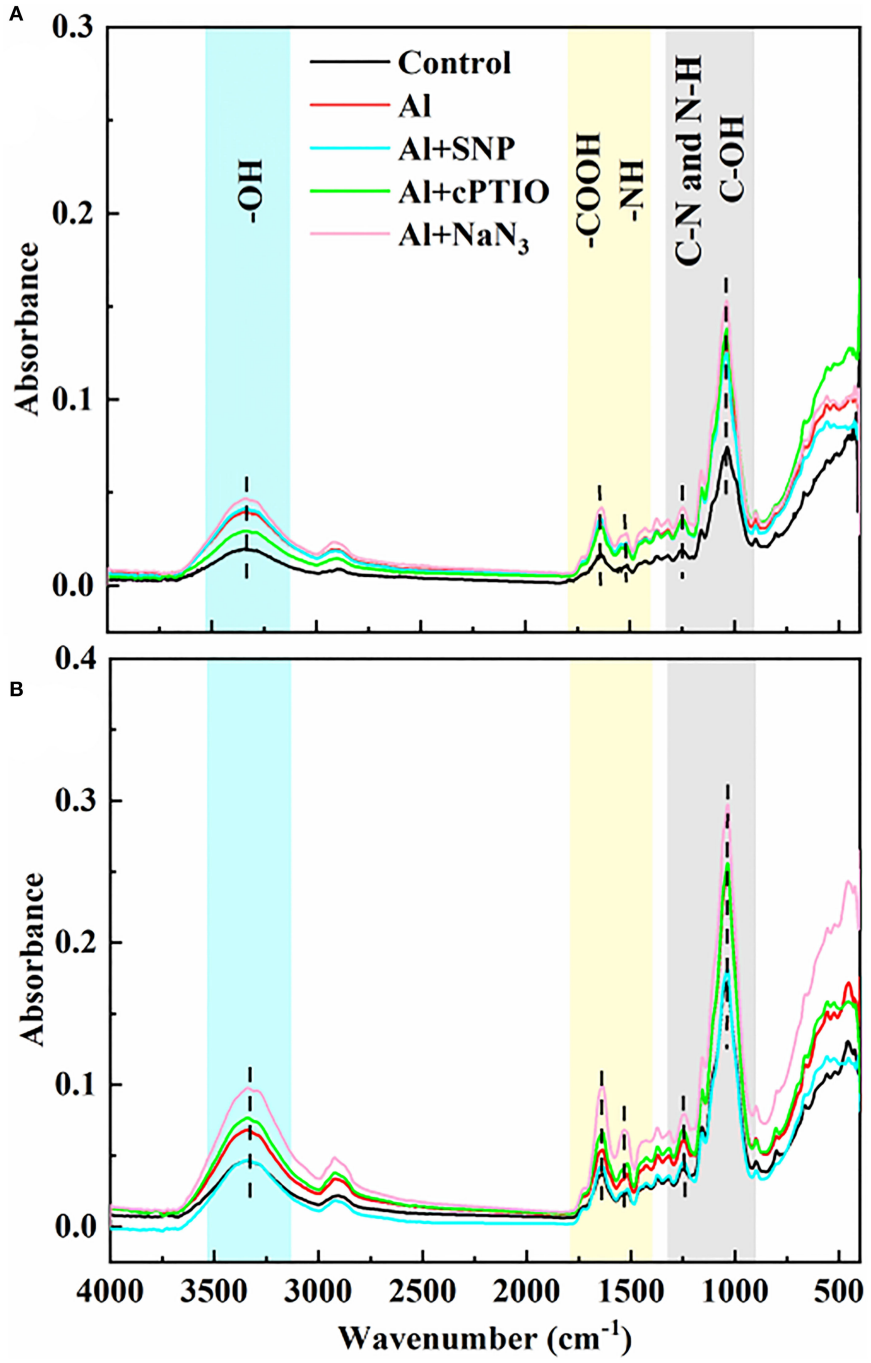
The effect of exogenous NO and NO scavengers on FTIR spectra of cell wall in **(A)** Nipponbare and **(B)** Zhefu 802 root tips under Al toxicity.

To detect the effects of exogenous NO on functional groups in the cell wall of rice root tips, FTIR analysis was used. The intensity of absorption peak in the spectrum represents the number of functional groups. As shown in Figure 3, the gradual peaks at 3,400, 1,635, 1,543, 1,247, and 1,031 cm^−1^ can be attributed to the polysaccharides (-OH stretching vibration), amide I (antisymmetric stretching vibrations of carboxyl), amide II (N-H bending vibrations), amide III (C-N stretching and N-H bending vibrations), and hemicelluloses (C-OH bending vibrations) (Xu et al., 2015; Liu et al., 2017; Ren et al., 2020). Under Al treatment, the intensity of characteristic peaks on the cell walls of both genotypes was significantly increased, indicating that Al induced the increase in the number of functional groups on the cell walls. Compared with the Al treatment, the addition of SNP significantly reduced the absorption peak intensity at 3,400, 1,635, 1,543, and 1,031 cm^−1^ in the cell wall, suggesting the ionizable groups of carboxyl, amide, and hydroxyl deceased. The addition of cPTIO and NaN_3_ reversed this result, the absorb peaks of these function groups moved to a higher spectra peak. The changed characteristic spectra of functional groups' spectra peaks were benefitted from the capability of Al binding, implying NO modified the number of negatively charged functional groups on the cell wall, thus reducing Al accumulation.

### Effect of BFA and SNP on root growth and Al content in root tips and cell wall

**Figure 4:**
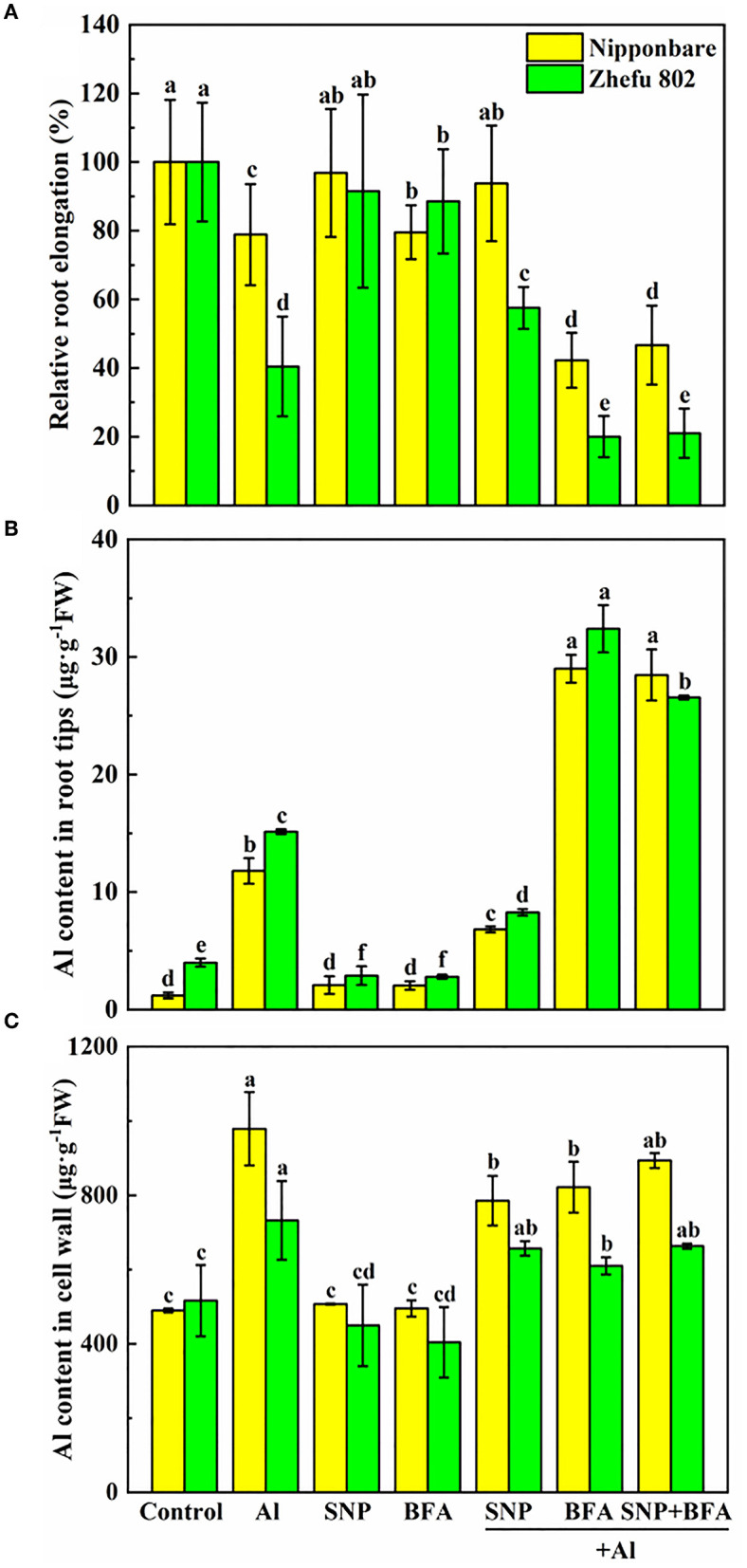
Effect of BFA and SNP on **(A)** rice root growth, **(B)** Al content in rice root tips, and **(C)** cell wall Al content. Columns with different letters are significantly different at *P* < 0.05.

We added an inhibitor of vesica secretion to identify the role of NO on cell wall structure and composition. Under Al toxicity, SNP significantly reduced Al content in root tips and cell wall (Figures 4B,C), and increased the relative root elongation (Figure 4A), indicating SNP alleviated Al toxicity. While BFA significantly inhibited root growth and decreased Al content in cell wall of root tips. Furthermore, there is no significant effect on root growth, Al content in root tips and cell wall between BFA + Al and SNP + BFA + Al treatment. These results suggested that the inhibition of cell wall components greatly reduces the Al which binding in the cell wall, but increases Al entering into the plasma membrane and exacerbates the Al toxicity of rice.

### Effect of BFA and SNP on cell wall composition under Al toxicity

The addition of BFA affects the content of cell wall polysaccharides. Compared with Al treatment, the contents of pectin and hemicellulose 1 and 2 in the cell wall significantly decreased cotreated by BFA and Al. And the cell wall pectin content of Nipponbare and Zhefu 802 also decreased by 56.64 and 50.33% in SNP + Al treatment, while the hemicellulose 1 and hemicellulose 2 contents showed no significant difference compared with the group treated with Al alone. In the case of BFA and Al treatment at the same time, the addition of SNP had no significant effect on the content of each component in the cell wall. It shows that NO doesn't change the blocking effect of BFA on cell wall components.

### BFA and NO modified the number of functional groups in the cell wall of rice root

**Figure 5:**
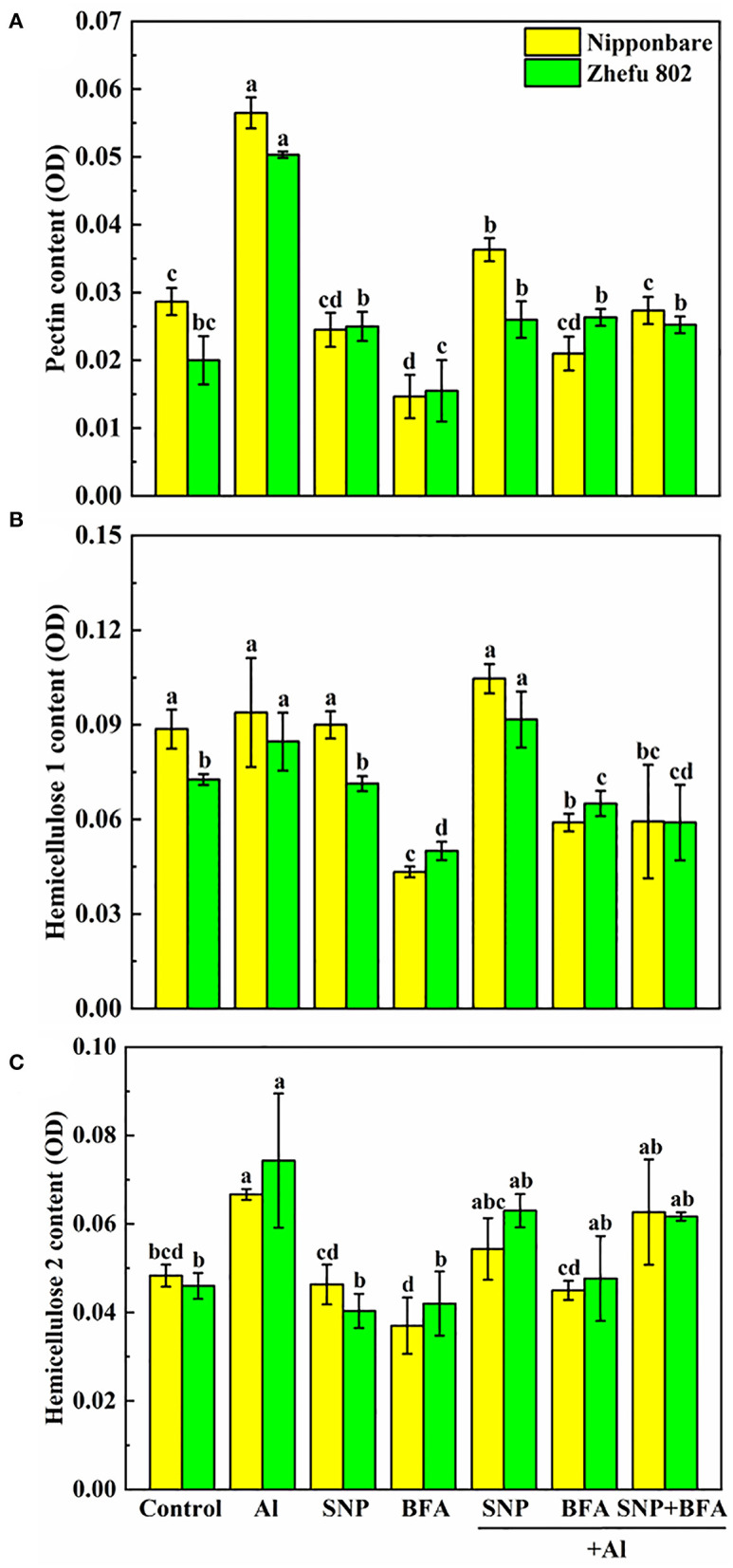
Effect of BFA and SNP on cell wall composition in rice root tips **(A)** pectin content, **(B)** hemicellulose 1 content, and **(C)** hemicellulose 2 content. Columns with different letters are significantly different at *P* < 0.05.

**Figure 6:**
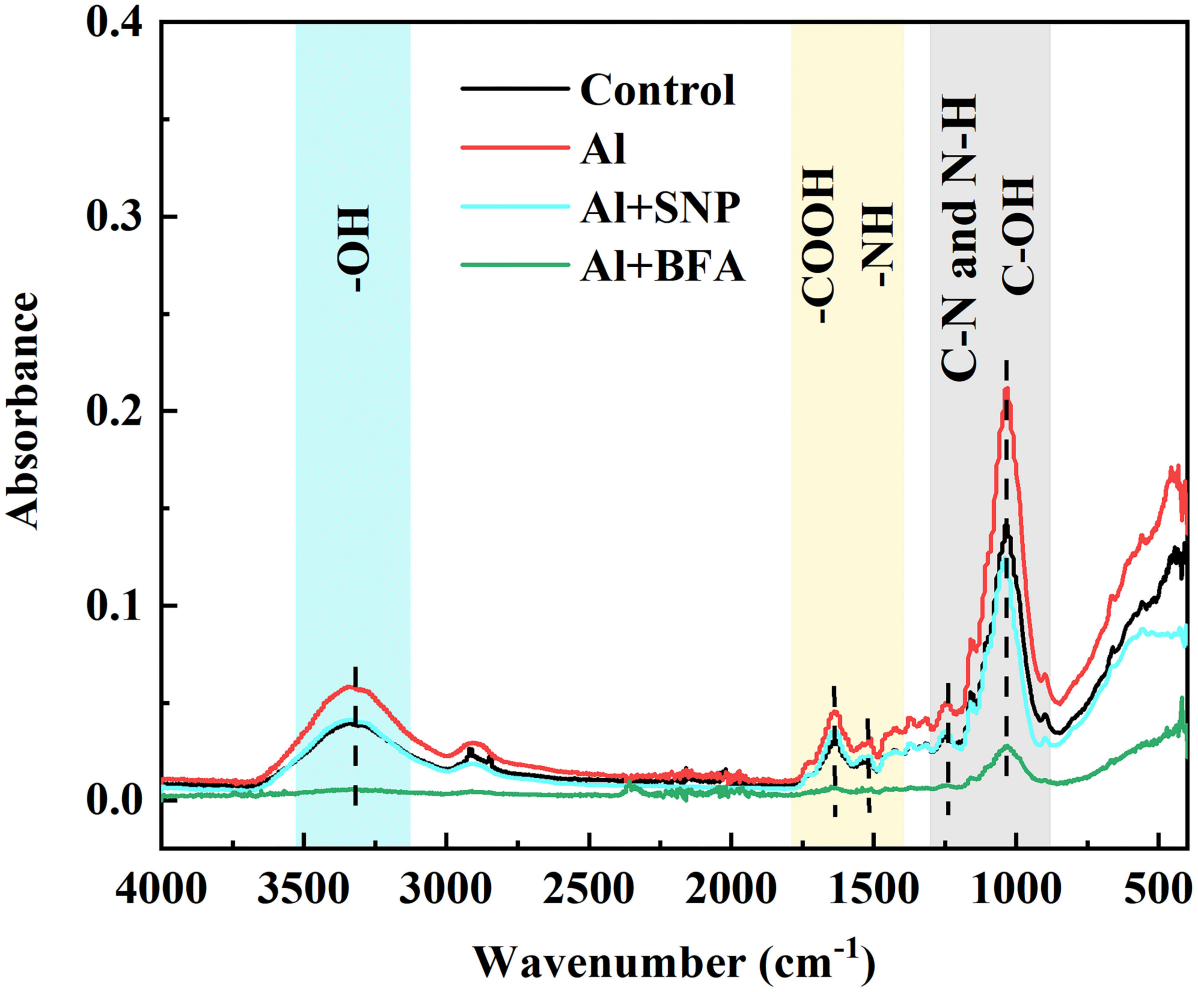
The effect of BFA and SNP on FTIR spectra of cell wall in root tips under Al toxicity.

We compared the differences of functional groups on the root cell wall of Al and Al + BFA treatment groups by FTIR (**Figure 6**. Contrasted with the Al treatment group, the addition of BFA significantly reduced the absorption peak intensity at 1,031 cm^−1^. However, the signal intensity of the absorption peak at 3,320, 2,902, and 1,635 cm^−1^ become very weak. The change of these functional groups is related to the change of cell wall components such as pectin and hemicellulose. This is consistent with the reduction in pectin and hemicellulose content due to BFA in Figure 5.

## Discussion

Al toxicity is the most vital soil factor restricting plant growth and development in acidic soil (Riaz et al., 2018). The toxic effects of Al on plant growth, cell wall biosynthesis and structure have been clarified in plants such as rice (Ranjan et al., 2021a). Studies in recent years have shown that NO plays a pivotal role in tolerance to kinds of abiotic or biotic stresses (Bhuyan et al., 2020). According to previous studies, exogenous NO can effectively slow down the damage of Al by enhancing Al exclusion from the root tips through modulating cell wall composition and structure, and decreasing ROS accumulation (Pan et al., 2017; Faria-Lopes et al., 2019; Tiwari et al., 2019). These results indicated that NO may be an important signal regulator mediating the Al toxicity response. However, the direct evidence needs to be studied. In our study, the supply of SNP significantly reduced Al content in root tips and cell walls (Figures 4B,C), and also decreased H_2_O_2_, O^2−^, and MDA accumulation (Figure 2) in roots treated with Al. The results were similar to the previous studies (Chauhan et al., 2021). The application of cPTIO and NaN_3_ significantly intensified the negative effects in root growth, and increased the mononuclear Al content (Figures 1B,C) and ROS accumulation (Figure 2) under 50 μM Al toxicity, further proving that NO is effective to alleviate Al toxicity.

BFA is an inhibitor of the endoplasmic reticulum–Golgi secretory pathway, usually acting on guanine nucleotide exchange factors in ARF proteins required for the formation of transport vesicles envelope, and BARs proteins that link Golgi structural integrity (Li et al., 2019). Keidan's results showed that BFA inhibited the accumulation of soluble polysaccharides in the cell wall, namely, uronic acid and mannose (Keidan and Friedlander, 2009). Exogenous addition of BFA can promote the accumulation of starch granules in cells, which may be caused by inhibiting the secretion of Golgi apparatus and the accumulation of polysaccharides in cells (Zhang et al., 2021a). Therefore, BFA treatment blocks the transport of cell wall materials, thus making the cell wall unable to properly synthesize due to a lack of cell wall materials, which leads to a significant reduction in pectin and hemicellulose content (Figure 5). The FTIR spectrum also proves this result, because the characteristic peaks of many functional groups disappear after BFA treatment (Figure 6). In addition, as cell wall synthesis is blocked, the function of the cell wall becomes dysfunctional, then a large amount of Al is absorbed and accumulated by rice roots, aggravating the toxic effect of Al (Figure 4B), suggesting that the integrity of cell wall mediates plant growth and Al tolerance. Because of the presence of BFA, SNP loses the effect of alleviating Al toxicity and cannot reverse the reduction of pectin and cellulose content caused by BFA (Figures 4, 5), suggesting that the regulation of NO on cell wall biosynthesis is downstream of the transportation and biosynthesis regulated by BFA.

In addition to the biosynthesis of cell wall components, the assembly of the cell wall, organizing the wall polymers into the functional structure, is also important for cell wall function construction (Hummel et al., 2010). Methyl esterification with the chemical groups of methyl is a major modification for pectic polymers, and acetylation with acetyl is also another major modification for hemicellulosic polymers (Zhu et al., 2014; Zhang et al., 2022). FTIR is a convenient and accurate technique to analyze the changes in the cell wall structure. Zhang et al. showed that Al downregulated the esterified and non-esterified carboxyl groups of pectin at the absorbance at 1,740 and 1,649 cm^−1^, making more Al bind to citrus lateral root cell wall through FTIR technology (Zhang et al., 2022). The addition of Boron (B) significantly reduced the peak values associated with pectin and hemicellulose under Al toxicity, suggesting that B modified cell wall composition and structural changes and accumulated into the plasmid caused by Al (Riaz et al., 2018). B also reduced the toxic effect of Cd in rice by promoting the exposure of -COOH and -OH on root cell walls, making more Cd chelate to the cell walls (Riaz et al., 2021). Recent FTIR spectroscopy studies showed that BFA treatment makes all functional groups at a low level (Figure 6). Compared with Al treatment, SNP significantly decreased absorption peaks at 3,400, 1,635, 1,543, and 1,031 cm^−1^ in the cell wall, which were produced by O-H, N-H, amide I, II, and III, and C=O, suggesting the ionizable groups of carboxyl, amide, and hydroxyl deceased (Figure 3). A decrease in the quantity of these negatively charged functional groups is equivalent to a decrease in the Al binding sites. These functional groups are also easy to be oxidized, which reduces the degree of oxidative stress in cells. Therefore, NO not only regulates the components of pectin but also modifies cell wall structure by regulating the proportion of cell wall functional groups. Our previous study showed that SNP reduced PME activity (Lan et al., 2021), the same results were reported by Zhang et al. (2011), Sun et al. (2016), and Yang et al. (2018). Yang et al. (2018) further proved that SNP can inhibit Al-activated PME activity and gene expression from the molecular level (Correa-Aragunde et al., 2008). Guo et al. and Zhao et al. reported that NO treatment reduces the activities of PME, polygalacturonase, transeliminase, and cellulase, thus delaying the softening of the cell wall (Guo et al., 2014; Zhao et al., 2019). The reason is that NO is transferred to the sulfhydryl group of the cysteine residue of the target protein by S-nitrosylation, which acts as a central regulator of post-translational modification of the protein (Moro et al., 2017). Furthermore, NO regulated the transcription level of cellulose synthase (CESA) in roots and thus regulates cellulose synthesis. So, we speculated that NO might modify the structure downstream biosynthesis and composition of the cell wall, strengthen the polysaccharide link and diverse functions of the cell wall, leading to normal plant growth and reproduction.

## Conclusions

In conclusion, this study reveals that BFA exacerbates Al toxicity in rice by blocking the biosynthesis of cell wall polysaccharide including pectin and two kinds of hemicellulose, then enhancing the accumulation of Al in rice root tips. NO application significantly decreases the content of pectin polysaccharide and modifys functional groups of hydroxyl, carboxyl, and amino in the cell wall. While NO cannot change the inhibition of BFA-induced polysaccharide biosynthesis and root growth. NO might modify cell wall partial composition and their functional groups, which alleviates Al toxicity.

## Data availability statement

The raw data supporting the conclusions of this article will be made available by the authors, without undue reservation.

## Author contributions

JY and JZhu wrote the whole manuscript. HS and KW conducted the experiment(s). JZhou analyzed the results. CX and MC revised the manuscript. All authors contributed to the article and approved the submitted version.

## Funding

This work was supported by the National Natural Science China (30800705 and 31101599), the Provincial Natural Science of Zhejiang (LY18C150007 and LY15C150004), and the Zhejiang Key Laboratory of Subtropic Soil and Plant Nutrition, China.

## Conflict of interest

The authors declare that the research was conducted in the absence of any commercial or financial relationships that could be construed as a potential conflict of interest.

## Publisher's note

All claims expressed in this article are solely those of the authors and do not necessarily represent those of their affiliated organizations, or those of the publisher, the editors and the reviewers. Any product that may be evaluated in this article, or claim that may be made by its manufacturer, is not guaranteed or endorsed by the publisher.
